# Editorial

**DOI:** 10.5152/tud.2023.270123

**Published:** 2023-01-01

**Authors:** Ateş Kadıoğlu

**Affiliations:** 1 Professor of Urology, University of Istanbul, Editor in Chief

Dear Colleagues,

It is my pleasure to introduce to you the Urology Research and Practice.

Urology Research and Practice carries on the legacy of the highly successful Turkish Journal of Urology, which has been continuously published since 1976. From its inception, the journal has held the leading position in the Turkish urology field and has been a remarkable example for good publishing practices in the country, courtesy of the Turkish Association of Urology.

At the end of 2022, Turkish Association of Urology decided to change the title of the journal to more accurately represent its ever-chang-

Urology Research and Practice’s mission to enhance its regional and global impact by presenting vital Turkish research in the field to an international audience, alongside international contributions, holds great importance for the Turkish Association of Urology.

To better support this mission, the editorial board of Urology Research and Practice has been expanded. The newly appointed section editors (Thomas Knoll, Roger Roman Dmochowski, Juan Gómez Rivas, Walid Farhat, and Ranjith Ramasamy) will provide enhanced international representation for the journal’s growing global authorship. Furthermore, the journal aims to recruit additional editorial board members in the coming weeks and months.

We remain committed to establishing Urology Research and Practice as a premier publication in the field of urology, by adhering to rigorous scientific and ethical standards. We extend our heartfelt appreciation to the editorial board members, peer reviewers, and authors who have submitted outstanding papers, for their invaluable contributions. With your assistance, we aim to progress in the new phase and achieve our objectives. We take pride in the opportunity to review the articles you submit to the journal and jointly contribute to the body of literature in this field.

